# Nomograms Incorporating the CNLC Staging System Predict the Outcome of Hepatocellular Carcinoma After Curative Resection

**DOI:** 10.3389/fonc.2021.755920

**Published:** 2022-01-21

**Authors:** Rui Liao, Xu-Fu Wei, Ping Che, Kun-Li Yin, Lei Liu

**Affiliations:** ^1^ Department of Hepatobiliary Surgery, The First Affiliated Hospital of Chongqing Medical University, Chongqing, China; ^2^ Department of Urology, The First Affiliated Hospital of Chongqing Medical University, Chongqing, China; ^3^ Department of Pediatric Surgery, Maternity and Child Health Hospital of Chongqing Hechuan, Chongqing, China

**Keywords:** hepatocellular carcinoma, China liver cancer staging system, nomogram, surgery, recurrence, prognosis

## Abstract

**Purpose:**

Prediction models of postoperative outcomes of patients with hepatocellular carcinoma (HCC) after surgery based on the China liver cancer (CNLC) staging system are rare. This study aimed to compare the prognostic abilities of CNLC, Tumor-Node-Metastasis (TNM) 8th edition, and Barcelona Clinic Liver Cancer (BCLC) staging systems for HCC after curative resection. We developed two nomograms incorporating the CNLC staging system to predict the postoperative recurrence-free survival (RFS) and overall survival (OS) of HCC patients.

**Patients and methods:**

The prognostic abilities of the CNLC, TNM and BCLC staging systems for HCC after curative resection were compared using receiver operating characteristic (ROC) curves. Two nomograms incorporating five selected risk factors were constructed based on multivariate Cox regression in the primary cohort of 312 HCC patients. It was validated with an independent validation cohort of 130 HCC patients. The predictive performance and discrimination ability of the two nomograms were further evaluated and compared with those of the TNM and BCLC staging systems.

**Results:**

The CNLC staging system had a higher area under the receiver operating characteristic curve (AUROC) value for both OS (AUC=0.692) and RFS (AUC=0.673) than the TNM (ROC=0.667 for OS and 0.652 for RFS) and BCLC (ROC=0.671 for OS and 0.670 for RFS) staging systems. The independent predictors of OS (cirrhosis, gamma-glutamyl transpeptidase (GGT), tumor differentiation and CNLC staging system) and RFS (α-fetoprotein (AFP) and CNLC staging system) were incorporated into the two nomograms. The OS and RFS nomograms consistently outperformed the TNM and BCLC staging systems in the primary cohort. These results were verified in the validation cohort. In the 442 patients with HCC, the RFS nomogram could predict early recurrence very well.

**Conclusion:**

The two proposed nomograms incorporating the CNLC staging system can predict the outcomes of patients with HCC after curative hepatectomy in clinical practice.

## Introduction

Hepatocellular carcinoma (HCC) is a malignant tumor mainly caused by hepatitis B (HBV) or C viral (HCV) infection and it accounts for the majority of primary liver cancers. Globally, the incidence of HCC is steeply rising, and currently, it ranks as the fourth most common cause of cancer-related death in 2018 with a notably poor prognosis (1). Unfortunately, most HCC patients are diagnosed at advanced disease stages and miss the opportunity for curative resection (e.g., hepatectomy and liver transplantation) (2). Even though curative therapies remain a treatment option available to some HCC patients, their long-term outcomes are still generally poor due to their high rate of tumor recurrence (3). Thus, it is of paramount importance to establish effective methods to stratify optimal candidates for curative surgery and individualize anticancer treatment response surveillance.

To date, a number of risk factors have been reported to predict the outcomes and prognosis of HCC. Among them, the severity of liver dysfunction, vascular invasion, tumor size and number, and the presence of metastases are considered to be the most important factors in determining survival (4). Currently, there are more than 15 clinical staging systems including these prognostic factors, such as the (1) American Joint Commission on Cancer (AJCC) seventh edition (5), (2) the Barcelona Clinic Liver Cancer (BCLC) system (6), (3) Cancer of the Liver Italian Program (CLIP) system (7), (4) Japan Integrated Staging Score (JIS) system (8), (5) Okuda staging system (9), (6) Vauthey’s system (10), (7) the albumin-bilirubin (ALBI) grading system (11), and (8) the Hong Kong Liver Cancer staging system (12). Although these staging systems could guide practitioners to the best options for therapeutic approaches, presently, a widely accepted optimal prognostic system is not available, particularly for surgical candidates.

Globally, approximately half of newly diagnosed HCC cases occur in China with an HBV infection background. In 2017, the China liver cancer (CNLC) staging system was established by Chinese experts according to recent HCC prognostic evidence, with subsequent modifications and updates for treatment allocations in 2019 (13–15). A recent comparative study (16) found that the BCLC system and the CNLC classification, as evidence-based staging systems and treatment algorithms, were useful in assisting treatment selection. Moreover, the CNLC staging system seems to perform better for HCC patients than the BCLC system. However, they often have a lower predictive ability than that of genuine prognostic scores due to structural variables not prognostically considered in real-life populations. Therefore, they result in a suboptimal prognostic performance (C index<0.7), suggesting that some key factors need to be incorporated into these systems to achieve substantial improvements for the prognostic estimation of HCC patient outcomes (16).

Compared to traditional staging systems, we have developed several nomograms for predicting the survival and recurrence of HCC that showed higher prognostic power than traditional staging systems (e.g., BCLC, TNM, etc.) (17–20). In this study, we compared the prognostic performance of some key risk variables and set up two reliable nomograms incorporating the CNLC staging system, and they could provide more accurate estimations of the prognosis of patients with HCC.

## Materials and Methods

### Patients and Study Design

The patients enrolled in the study were from the First Affiliated Hospital of Chongqing Medical University between January 2014 and December 2015. In this retrospective study, a total of 531 consecutive patients were pathologically diagnosed with primary HCC and underwent curative resection. Eighty-nine patients were excluded according to the inclusion and exclusion criteria: (1) all patients had valid and reliable laboratory test data; (2) no preoperative extrahepatic metastases; (3) no anticancer treatments before the operation; (4) mayor R0 curative resection of all tumor nodules; and (5) complete patient records and follow-up data. Finally, 442 patients qualified for this study as a primary cohort (January 2014 to June 2015, n=312) to develop the nomograms and a validation cohort (July 2015 to December 2015, n=130). This study was performed in compliance with the 1975 Helsinki Declaration and was approved by the Ethics Review Committee of the First Affiliated Hospital of Chongqing Medical University. Informed consent to participate in this study was obtained from the research subjects prior to study commencement. The study participants also gave consent to have their data published.

### Follow-Up

After discharge from the hospitals, all patients underwent follow-up every 3 months in the first 2 years and every 6 months afterward until signs of recurrence emerged over the next 3 to 5 years. During each regular surveillance for recurrence, serum α-fetoprotein (AFP), serum biochemistry, abdomen ultrasonography and chest and abdominal computed tomography (CT) examinations were conducted. Patients with recurrence received further treatment, including a second liver resection, radiofrequency ablation, transcatheter arterial chemoembolization or symptomatic treatment, according to the tumor size, site, number, hepatic functional reserve, extent of disease and general health of the patient. Recurrence-free survival (RFS) was defined as the interval between the date of surgery and recurrence. Overall survival (OS) was defined as the interval between the date of surgery and death or the last follow-up. Recurrence was subdivided into early (≤ 24 months) and late recurrence (> 24 months) (17).

### Prognostic Nomograms

The two nomograms were built based on the results of the multivariable analyses of RFS and OS in the primary cohort. Tumor number, tumor size and vascular invasion were not included in the nomograms because they are structural variables of the CNLC staging system, which was incorporated into the two nomograms. The final model was determined by a backward step-down selection process. Discrimination was evaluated by calculating the C-index. The values of the C-index were used to assess the discrimination ability (0.5–1.0). Calibration plots were used to compare the predicted survival by the Kaplan–Meier curves of the quartiles of predictions. Bootstraps with 1000 resamples were used for both the validation of the nomograms and for calibration assessment (17–20).

### Statistical Analysis

Statistical analyses were performed using SPSS 24.0 (SPSS, Inc., Chicago, IL, USA) and the rms package in R version 3.4.0 (http://www.r-project.org/). The *χ* ^2^ test or Fisher’s exact test was used to compare the categorical variables. Continuous variables were compared using Student’s t-tests with a normal distribution or nonparametric Mann–Whitney U-tests with an irregular distribution and reported as the mean ± standard deviation (SD). The sensitivity and specificity were defined by receiver operating characteristic (ROC) curves. Pearson’s or Spearman’s ρ coefficient tests were used to analyze the correlation between variables. RFS and OS curves were calculated by Kaplan–Meier survival estimates and compared using the log-rank test. Factors found to be significant were subsequently enrolled in the multivariable Cox proportional hazard regression models.

## Results

### Baseline Characteristics

The clinical baseline characteristics of the 442 HCC patients in the primary and validation cohorts are described in **Table 1**. Both cohorts were mostly comprised of men (84.6% and 85.4%) and were similar in age composition. Moreover, the majority of the patients were HBsAg positive (85.6% and 88.5%) and had cirrhosis (86.9% and 93.1%). Vascular invasion occurred in 44.2% and 55.8% of patients, and the median tumor sizes were 5.5 and 4.0 cm in the primary cohort and the validation cohort, respectively.

**Table 1:** Baseline of Patient Characteristics.

Characteristics	Primary cohort	Validation cohort	P-value
	n = 312	n = 130	
Age, yr, median, (range)	51.5 (18-80)	51.5 (25-78)	0.512
Gender (Female/Male)	48/264 (15.4%/84.6%)	19/111 (14.6%/85.4%)	0.533
Cirrhosis (yes/no)	271/41(86.9%/13.1%)	121/9 (93.1%/6.9%)	0.060
GGT, U/L, median (range)	64.0 (8.0-811.0)	61.0 (13.0-513.0)	0.252
ALB, g/L, median (range)	42.0 (28.0-53.0)	43.0 (31.0-54.0)	0.335
TBIL, µmol/L, median (range)	15.4 (4.4-75.2)	10.75 (4.5-34.4)	<0.001
AFP, ng/ml, median (range)	132.0 (0-60500.0)	130.0 (0-60500.0)	0.223
HBsAg (Positive/Negative)	267/45 (85.6%/14.4%)	115/15 (88.5%/11.5%)	0.420
Tumor number (single/multiple)	229/83 (73.4%/26.6%)	117/13 (90.0%/10.0%)	<0.001
Vascular invasion (yes/no)	138/174 (44.2%%/55.8%)	37/93 (28.5%/71.5%)	0.002
Tumor differentiation (I-II/III-IV)	174/138 (55.8%/44.2%)	95/35 (73.1%/26.9%)	0.001
Tumor size, cm, median (range)	5.5 (0.9-23.0)	4.0 (1.5-21.0)	0.002
BCLC stage (0-A/B/C)	136/35/141 (43.6%/11.2%/45.2%)	57/34/39 (43.8%/26.2%/30%)	0.009
TNM stage (I/II/III)	139/101/72 (44.6%/32.4%/23.0%)	85/42/3 (65.4%/32.3%/2.3%)	<0.001
CNLC stage (Ia/Ib/IIa/IIb/IIIa)	54/93/26/4/135 (17.3%/29.8%/8.3%/1.3%/43.3%)	56/27/8/3/36 (43.0%/20.8%/6.2%/2.3%/27.7%)	<0.001

GGT, gamma-glutamyl transpeptidase; ALB, Albumin; TBIL, total bilirubin; AFP, alpha fetoprotein; HBsAg, hepatitis B virus surface antigen; BCLC, Barcelona Clinic Liver Cancer; TNM, Tumor-Node-Metastasis; CNLC, China liver cancer staging system.

### OS and RFS in the Two Cohorts

The median follow-up was 54.0 months for the entire cohort, 55.5 months for the primary cohort, and 50.5 months for the validation cohort. In the primary cohort, the median OS and RFS were 36.5 (range, 1.0–81.5 months) and 34.0 months (range, 1.0–78.5 months), respectively. The 1-, 3-, and 5-year OS rates were 82.2%, 68.4% and 43.5%, respectively. The 1-, 3-, and 5-year RFS rates were 70.6%, 51.4% and 33.3%, respectively. In the validation cohort, the median OS and RFS were 37.1 (range, 1.0–65.0 months) and 32.5 months (range, 1.0–64.5 months), respectively. The 1-, 3-, and 5-year OS rates were 80.8%, 65.4% and 42.3%, respectively. The 1-, 3-, and 5-year RFS rates were 67.1%, 47.4% and 31.8%, respectively.

### Prognostic Abilities of the CNLC, BCLC and TNM Staging Systems

**Figure 1:**
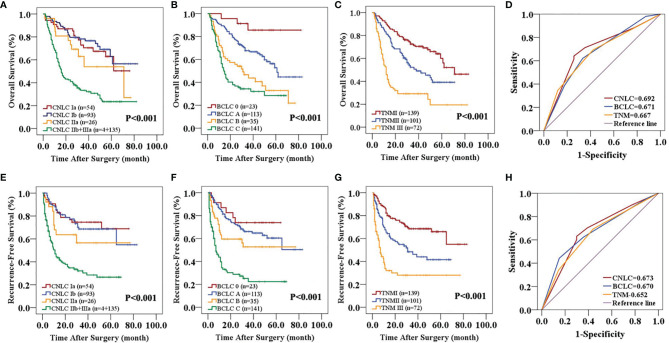
Predictive accuracy comparison of the three staging systems for the prognosis of patients with HCC in the primary cohort. CNLC **(A, E)**, BCLC **(B, F)** and TNM 8th edition **(C, G)** staging systems are associated with OS **(A–C)** and RFS **(E–G)** of HCC after curative resection. ROC curves were used to compare the predictive accuracy of the three staging systems for assessing OS **(D)** and RFS **(H)** rates in the primary cohort.

**Figure 2:**
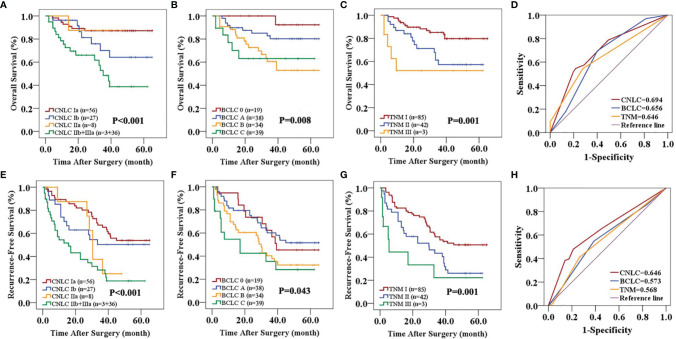
Predictive accuracy comparison of the three staging systems for the prognosis of patients with HCC in the validation cohort. CNLC **(A, E)**, BCLC **(B, F)** and TNM 8th edition **(C, G)** staging systems are associated with OS **(A–C)** and RFS **(E–G)** of HCC after curative resection. ROC curves were used to compare the predictive accuracy of the three staging systems for assessing OS **(D)** and RFS **(H)** rates in the validation cohort.

In the primary cohort, the CNLC, BCLC and TNM staging systems all predicted the OS (P<0.01) and RFS (P<0.01) of patients with HCC after curative resection (**Figures 1A–C, E–G**). Receiver operating characteristic (ROC) curve analyses showed that the CNLC staging system (ROC=0.692 for OS and 0.673 for RFS) performed better for HCC patients than the BCLC (ROC=0.671 for OS and 0.670 for RFS) and TNM (ROC=0.667 for OS and 0.652 for RFS) staging systems (**Figures 1D, H**). The validation cohort had similar data (**Figure 2**), which is consistent with Vitale’s recent report (16).

### Independent Prognostic Factors in the Primary Cohort

In univariate analyses (**Table 2**), gamma-glutamyl transpeptidase (GGT, P<0.001 and =0.020), alpha-fetoprotein (AFP, both P<0.001), tumor number (P=0.002 and <0.001), vascular invasion (both P<0.001), tumor differentiation (P<0.001 and =0.020), tumor size (both P=0.001) and CNLC staging system (both P<0.001) were identified as significant prognostic factors for OS and RFS in the primary cohort, respectively. Both cirrhosis (P=0.004) and albumin (ALB, P=0.02) could predict OS alone. Tumor number, tumor size and vascular invasion were not included in subsequent multivariable and nomograms because they are structural variables of the CNLC staging system. Multivariable (**Supplementary Table 1**) analyses demonstrated that cirrhosis (P=0.016), GGT (P=0.002), tumor differentiation (P<0.001) and the CNLC staging system (P<0.001) were independent prognostic factors of OS. Moreover, AFP (P=0.017) and the CNLC staging system (P<0.001) were related to RFS.

**Table 2:** Multivariate Analysis of OS and RFS of HCC in primary cohort.

Factors	OS		RFS	
	HR (95%CI)	P-value	HR(95%CI)	P-value
AFP (>20/≤20 ng/ml)	–	NS	1.615 (1.089-2.396)	0.017
Cirrhosis (yes/no)	2.137 (1.154-3.956)	0.016	–	NA
GGT (>64/≤64 U/L)	1.222 (1.078-1.384)	0.002	–	NS
ALB (>42/≤42 g/L)	–	NS	–	NA
Tumor differentiation (I-II/III-IV)	1.776 (1.287-2.449)	<0.001	–	NS
CNLC (Ia/Ib/IIa/IIb/IIIa)	1.450 (1.303-1.614)	<0.001	1.424 (1.273-1.592)	<0.001

Multivariate analysis, Cox proportional hazards regression model. HCC, hepatocellular carcinoma; OS, Overall survival; RFS, Recurrence-free survival; HR, hazard ratio; CI, confidence interval; AFP, alpha fetoprotein; GGT, gamma-glutamyl transpeptidase; ALB, Albumin; CNLC, China liver cancer staging; NS, not significance, NA, not adopted.

### Predictive Performance of the Nomograms

**Figure 3:**
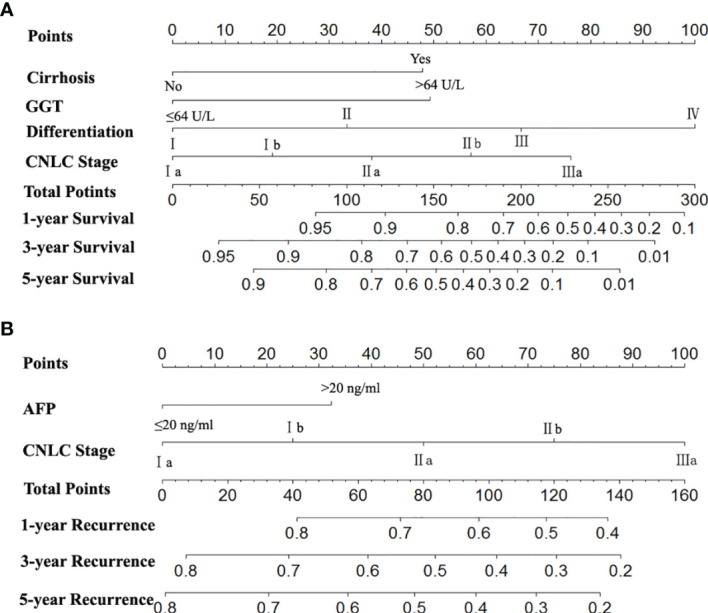
Nomograms for predicting survival and recurrence of HCC patients after surgery. To calculate the probability of OS **(A)** and RFS **(B)**, straight upward lines are drawn to determine the points accrued. The sum of these points is plotted on the total points bar to the probability to yield the 1-, 3-, and 5-year survival or recurrence rates.

**Figure 4:**
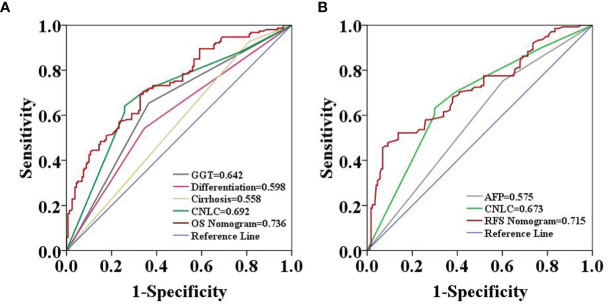
Predictive accuracy comparison of each variable included in the OS **(A)** and RFS **(B)** nomograms by ROC curve analyses in the primary cohort. The ROC curves showed that the two nomograms were superior to the other variables in predictive accuracy.

**Figure 5:**
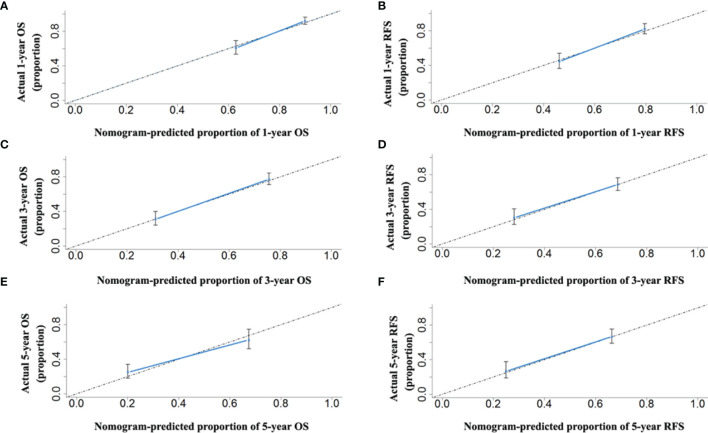
The calibration curves for predicting the 1-, 3- and 5-year OS **(A, C, E)** and RFS **(B, D, F)** rates by nomogram prediction and actual observation in patients with HCC in the primary cohort.

The two prognostic nomograms comprised the CNLC staging system and several other independent OS and RFS prognostic factors were derived from the primary cohort (**Figure 3**). The C-indices of the OS and RFS nomograms were 0.743 (95% CI: 0.707–0.779) and 0.701 (95% CI: 0.659–0.739), respectively, which were higher than those of the CNLC staging system (C-index: 0.665 for OS and 0.676 for RFS) (**Supplementary Table 2**). Similarly, the OS and RFS nomograms showed the largest AUROCs (0.736 for OS and 0.715 for RFS) (**Figures 4A, B**) compared with the CNLC staging system. The results suggest that the two nomograms had more accurate OS and RFS prognostic power than the CNLC staging system in patients with HCC after curative hepatectomy. The OS and RFS probability calibration plots showed acceptable overall consistency between the two nomograms for predictions and actual observations in the primary cohort at 1, 3 and 5 years after surgery (**Figure 5**).

### Validation of the Nomograms

**Figure 6:**
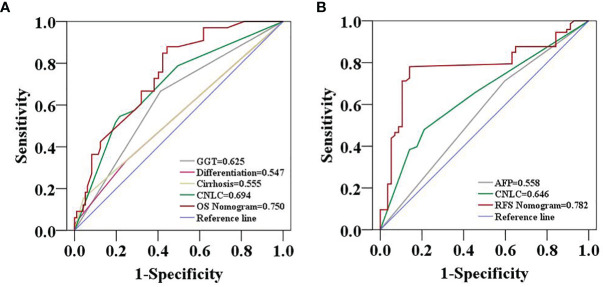
Predictive accuracy comparison of each variable included in the OS **(A)** and RFS **(B)** nomograms by ROC curve analyses in the validation cohort. The ROC curves showed that the two nomograms were superior to the other variables in predictive accuracy.

**Figure 7:**
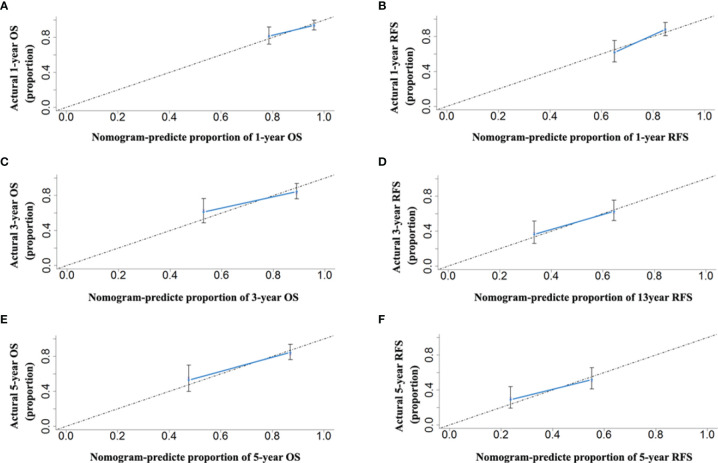
The calibration curves for predicting the 1-, 3- and 5-year OS **(A, C, E)** and RFS **(B, D, F)** rates by nomogram prediction and actual observation in patients with HCC in the validation cohort.

Comparing the tumor characteristics, although some patient demographics in the validation cohort were different from those in the primary cohort, the nomograms still had powerful predictive abilities for the HCC patients in the validation cohort. The C-indices of the nomograms for predicting OS and RFS were 0.739 (95% CI: 0.656–0.822) and 0.672 (95% CI: 0.641–0.703), respectively. The C-indices of OS and RFS for the CNLC staging system were 0.687 (95% CI: 0.592–0.780) and 0.650 (95% CI: 0.591–0.709), respectively. The ROC analyses showed that the two nomograms had larger AUCs (0.750 for OS and 0.782 for RFS) than the CNLC staging system (0.694 for OS and 0.646 for RFS, **Figure 6**). Both the OS and RFS probability calibration plots had good agreement between predictions and observations in the probability of 1-, 3- and 5-year recurrence and survival (**Figure 7**).

### The Predictive Performance of the RFS Nomogram for Early Recurrence

**Figure 8:**
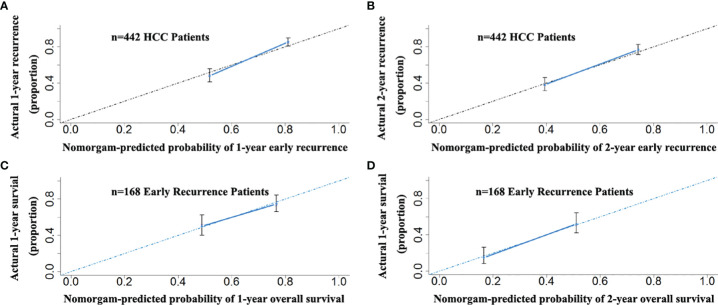
The calibration curves for the probability of 1- and 2-year RFS nomogram showed good agreement between prediction and observation in the probability of early recurrence in the total of 442 patients with HCC **(A, B)**. The calibration curves for the probability of 1- and 2-year OS nomogram showed good agreement between prediction and observation in the probability of overall survival in 168 early recurrence patients with HCC **(C, D)**.

In the 442 patients with HCC, there were 168 patients (123 and 45 patients in the primary and validation cohorts, respectively) with early recurrence (ER, ≤24 months). The RFS nomogram could predict early recurrence very well. The C-indices were 0.699 (95% CI: 0.652–0.710). Of the 168 patients with ER, the proposed nomogram also performed well for OS prediction. The C-index was 0.707 (95% CI: 0.645–0.749). The calibration curves for the probability of both RFS in the 442 patients at 1 and 2 years (**Figures 8A, B**) and OS in the 168 patients with ER (**Figures 8C, D**) fit well and suggested that the two proposed nomograms could be applied for the prediction of the OS of HCC patients with ER.

## Discussion

Current unmet clinical needs for HCC patients involve accurate staging, prognosis, and treatment allocation. A variety of staging systems have been proposed to reflect the oncological prognosis and to guide treatment decisions (21–23). To date, no consensus has been achieved on which one is the most appropriate paradigm to accurately predict the patient’s outcomes and to determine the appropriate intervention, particularly for surgical candidates with HCC (24). In this study, we identified and then compared the prognostic abilities of the CNLC with the BCLC and TNM staging systems, two commonly used staging systems, by ROC analysis and the C-index. Although the CNLC staging system performed better for HCC patients than the BCLC and TNM staging systems after curative resection, all three of them had a suboptimal prognostic performance (C-index<0.7), which suggested that some critical risk factors may not be included in these systems, and better paradigms with highly stable predictive accuracy for surgical HCC patients are needed. Different from many other nomograms constructed based on the combination of some molecules/genes and tumour characteristics, the main component of the nomograms, CNLC has been applicated widely in clinical practice, to a certain extent improving their reliabilities.

In addition to the CNLC staging system, the two nomograms integrated three independent risk factors for OS, including cirrhosis, GGT and tumor differentiation, and integrated AFP for predicting RFS. These risk factors have been identified previously for the surgical prognosis of HCC (25–30). First, the majority of HCC cases occur in a setting of cirrhosis, which constitutes an extremely heterogeneous inflammatory microenvironment and promotes the proliferation of premalignant cells and HCC development (29). Second, GGT can give rise to pro-oxidant reactions that can induce endogenous reactive oxygen species in tumor cells, which are involved in tumor formation, cell proliferation and apoptosis (26, 31, 32). Moreover, some inflammatory cytokines can induce the production of GGT, and GGT has prognostic effects on HCC development (26). Third, good differentiation to poor differentiation evolution is a critical phenomenon during HCC progression that is potentially related to the prognosis of HCC. In HCC tissues, poor tumor differentiation is significantly associated with reduced expression levels of the RCAN1 isoform 4, which acts as a suppressor of HCC through regulation of the calcineurin-nuclear factor of activated T cells pathway (33). Makiko’s finding suggested that the switch of transferrin receptor (TFR) expression from TFR-2 to TFR-1, both iron metabolism-associated transmembrane transport iron protein receptors, is also related to HCC dedifferentiation (34). Fourth, AFP gene expression is associated with the carcinogenesis of HCC. Tumor relapse from intrahepatic metastasis or multicentric origin is accompanied by inconsistent serum AFP (35). On the other hand, high AFP levels are associated with a powerful tumor-host immune response (36) and increased invasive and metastatic abilities of tumor cells, one of the reasons for the high recurrence rate of HCC after surgery (37).

The OS and RFS nomograms might contribute to a significantly increased predictive accuracy due to incorporation of the CNLC staging system and several reliable independent risk factors. In this study, although the CNLC, BCLC and TNM staging systems had the ability to stratify patients after curative hepatectomy into different risk categories, the two nomograms seemed to have better predictive accuracy for survival and recurrence. Finally, ROC analysis, the C-index and the calibration curve showed that the OS and RFS nomograms integrating the CNLC staging system were superior to the CNLC staging system alone and better than the BCLC and TNM staging systems.

Clinically, it is still practically impossible to predict ER (≤ 24 months), which generally has a worse prognosis and is often considered to be the result of occult metastasis of the primary tumor (17). Our model is more powerful (C-index: 0.701, 0.659–0.739) for predicting HCC recurrence following curative hepatectomy than the BCLC and TNM staging systems. The RFS nomogram showed satisfactory predictive accuracy for recurrence within 2 years (ER) for all patients in the two cohorts. More interestingly, our findings highlight that the OS nomogram exhibited powerful predictive performance for patients with ER. The power of the prediction of the two nomograms was supported by the C-index and the calibration curve. These findings might shed light on an important association between the nature of the primary tumor, such as tumor size, tumor number and vascular invasions, and the ER of HCC. Additionally, AFP may be an important gene associated with the dissemination of primary HCC tumor cells. Satellites nodules have already been identified as a risk factor of ER. However, this risk factor is not taken into consideration in this study due to lack of complete relevant information. Further investigation should be completed in the future.

We acknowledge that limitations exist in the present study (1). This was a retrospective study at a single institute. A trend toward significance indicates a need for large-scale multicenter studies for prospective verification. (2) Given the background HBV infection of most patients (86.4%, 382/442), the nomograms might not be suitable for HCC patients with etiologies other than HBV infection. (3) The CNLC staging system was established based on Chinese patients. Because the etiology and ethnic background of patients with HCC are diverse, these nomograms may not be suitable for a Western population mainly infected by HCV. (4) Although CNLC staging system has been applicated widely in Chinese HCC patients recently. But this nomogram may be further modified to improve its predictive accuracy and credibility.

In conclusion, the two nomograms improved the survival and recurrence predictive ability over the modified CNLC staging system. This information might be of more help for clinicians to thoroughly prepare HCC patients with potential early recurrence risks following surgery.

## Data Availability Statement

The original contributions presented in the study are included in the article/**Supplementary Material**. Further inquiries can be directed to the corresponding author.

## Ethics Statement

The studies involving human participants were reviewed and approved by the ethics review committee of the First Affiliated Hospital of Chongqing Medical University. The patients/participants provided their written informed consent to participate in this study.

## Author Contributions

RL: funding acquisition, conceptualization, supervision, writing—review and editing. X-FW: data curation, formal analysis, funding acquisition, writing—original draft. PC: data curation, formal analysis, methodology, writing—original draft. K-LY and LL: formal analysis, methodology.

## Funding

This research was supported by Science and Technology Research Program of Chongqing Municipal Education Commission (no. KJQN201800416), and Basic and Advanced Research Project of Science and Technology Commission of Chongqing Municipality (no. cstc2018jcyjAX0162 and cstc2018jscx-msybX0133); Science and Health Joint Research Project of Chongqing Municipality (2020GDRC013 and 2021MSXM026).

## Conflict of Interest

The authors declare that the research was conducted in the absence of any commercial or financial relationships that could be construed as a potential conflict of interest.

## Publisher’s Note

All claims expressed in this article are solely those of the authors and do not necessarily represent those of their affiliated organizations, or those of the publisher, the editors and the reviewers. Any product that may be evaluated in this article, or claim that may be made by its manufacturer, is not guaranteed or endorsed by the publisher.
