# Supplementary material for: Nomograms Incorporating the CNLC Staging System Predict the Outcome of Hepatocellular Carcinoma After Curative Resection
Source: Front Oncol. 2022 Jan 21;11:755920. doi: 10.3389/fonc.2021.755920 (PMC8814341; doi:10.3389/fonc.2021.755920)
Supplement: Supplementary file 1 [file Table_1.docx]

**Table S1** **Univariate Analysis of OS and RFS of HCC in primary cohort**

| **Factors** | **OS** | | **RFS** | |
| --- | --- | --- | --- | --- |
|  | **HR (95%CI)** | **P-value** | **HR (95%CI)** | **P-value** |
| Age | 0.992 (0.978-1.005) | 0.166 | 0.732 (0.522-1.025) | 0.065 |
| Gender (Female/Male) | 0.860 (0.547-1.351) | 0.510 | 0.656 (0.389-1.104) | 0.106 |
| Cirrhosis (yes/no) | 2.385 (1.291-4.408) | **0.004** | 1.525 (0.891-2.608) | 0.117 |
| GGT (>64/≤64 U/L) | 1.171 (1.051-1.306) | **<0.001** | 1.075 (0.925-1.250) | **0.020** |
| ALB (>42/≤42 g/L) | 0.877 (0.637-1.208) | **0.002** | 0.932 (0.667-1.302) | 0.676 |
| TBIL (>15.5/≤15.5µmol/L) | 0.940 (0.684-1.291) | 0.701 | 0.819 (0.585-1.147) | 0.240 |
| AFP (>20/≤20 ng/ml) | 1.991 (1.373-2.887) | **<0.001** | 1.969 (1.335-2.905) | **<0.001** |
| HBsAg (Positive/Negative) | 1.230 (0.769-1.969) | 0.384 | 1.133 (0.705-1.821) | 0.602 |
| Tumor number (single/multiple) | 1.683 (1.199-2.362) | **0.002** | 1.923 (1.350-2.739) | **<0.001** |
| Vascular invasion (yes/no) | 3.493 (2.501-4.877) | **<0.001** | 3.340 (2.360-4.725) | **<0.001** |
| Tumor differentiation (Ⅰ-Ⅱ/Ⅲ-Ⅳ) | 1.782 (1.295-2.451) | **<0.001** | 1.467 (1.050-2.050) | **0.020** |
| Tumor size (>3.0/≤3.0 cm) | 2.002 (1.294-3.098) | **0.001** | 2.119 (1.331-3.375) | **0.001** |
| CNLC (Ⅰa/Ⅰb/Ⅱa/Ⅱb/Ⅲa) | 1.475 (1.326-1.639) | **<0.001** | 1.456 (1.304-1.626) | **<0.001** |

Abbreviations: OS: overall survival; RFS: recurrence-free survival; HCC: hepatocellular carcinoma; HR: hazard ratio; CI: confidence interval; GGT; gamma-glutamyl transpeptidase; ALB: albumin; TBIL: total bilirubin; AFP: alpha-fetoprotein; HBsAg: hepatitis B virus surface antigen, CNLC: China liver cancer staging system.
