# Supplementary material for: Nomograms Incorporating the CNLC Staging System Predict the Outcome of Hepatocellular Carcinoma After Curative Resection
Source: Front Oncol. 2022 Jan 21;11:755920. doi: 10.3389/fonc.2021.755920 (PMC8814341; doi:10.3389/fonc.2021.755920)
Supplement: Supplementary file 2 [file Table_2.docx]

**Table S2 The C-index of the predictors in nomograms and** clinical staging systems

| **Predictor** | **Overall survival** | | **Recurrence-free survival** | |
| --- | --- | --- | --- | --- |
|  | **C-index** | **95% CI** | **C-index** | **95% CI** |
| Nomogram | 0.743 | 0.707-0.779 | 0.701 | 0.659-0.739 |
| AFP | NA | - | 0.584 | 0.547-0.621 |
| Cirrhosis | 0.540 | 0.514-0.566 | NA | - |
| GGT | 0.626 | 0.590-0.662 | NA | - |
| Tumor differentiation | 0.596 | 0.552-0.640 | NA | - |
| CNLC stage | 0.665 | 0.624-0.706 | 0.676 | 0.635-0.717 |
| TNM stage | 0.641 | 0.567-0.715 | 0.655 | 0.618-0.692 |
| BCLC stage | 0.654 | 0.618-0.690 | 0.656 | 0.621-0.691 |

Abbreviations: AFP: alpha fetoprotein; BCLC: Barcelona Clinic Liver Cancer; TNM: Tumor-Node-Metastasis; NA: not adopted.
